# Supplementary material for: Complexome profiling of the Chlamydomonas psb28 mutant reveals TEF5 as an early PSII assembly factor
Source: Plant Cell. 2025 Mar 17;37(6):koaf055. doi: 10.1093/plcell/koaf055 (PMC12164590; doi:10.1093/plcell/koaf055)
Supplement: koaf055_Supplementary_Data [file koaf055_supplementary_data.zip › psb28_interactive.zip!/plots/intensity/qualityPlots/peptides/_Cre09.g407650.t1.2.html]

### Cre09.g407650.t1.2

Trivial name:   
SeqCoverage [median]: 12.20  
Mass: 20.27 kDa  
  
 

| Sequence | MissedCleavage | Mass [Da] | wtIntensity | muIntensity | Score |
| --- | --- | --- | --- | --- | --- |
| AIFTVLK | 0 | 790.50 | 0.00 | 851.60 | 67.03 |
| SDAEAEDPASIEAFRK | 1 | 1734.81 | 1005.14 | 2772.88 | 73.30 |
|  |  |  | 1005.1 | 3624.5 |  |
